# The COVID-19 Pandemic in Brazil: Some Aspects and Tools

**DOI:** 10.3390/epidemiologia2030018

**Published:** 2021-06-23

**Authors:** Pedro Rafael D. Marinho, Gauss M. Cordeiro, Hemilio Fernandes C. Coelho, Poliana C. Cabral

**Affiliations:** 1 Department of Statistics, Federal University of Paraíba, João Pessoa 58051-900, Brazil; pedro.rafael.marinho@gmail.com (P.R.D.M.); hemilio@gmail.com (H.F.C.C.); 2 Department of Statistics, Federal University of Pernambuco, Recife 50670-901, Brazil; gauss@de.ufpe.br; 3 Department of Nutrition, Federal University of Pernambuco, Recife 50670-901, Brazil

**Keywords:** Brazil, COVID-19, GAMLSS models, statistics, tools, web application

## Abstract

The article presents some aspects related to the COVID-19 pandemic in Brazil including public health, challenges facing healthcare workers and adverse impacts on the country’s economy. Its main contribution is the availability of two web applications for online monitoring of the evolution of the pandemic in Brazil and South America. The applications provide the possibility to download data in different formats, view interactive maps and graphs of the cumulative confirmed cases, deaths and lethality rates, in addition to presenting plots of moving averages for states and municipalities. The predictions about new cases and new deaths caused by COVID-19, in states and regions of Brazil, are also reported using GAMLSS models. The forecasts can be easily used by public managers for effective decision-making.

## 1. Introduction

In December 2019, a doctor called Li Wenliang, who lived in Wuhan, China, was one of the first who identified the virus COVID-19. This doctor died from the same coronavirus that he discovered. From that time on, the virus spread worldwide, and the World Health Organization (WHO) declared, on 11 March 2020, a pandemic. Because of large variations in testing, the COVID-19 points out to an underestimation in the number of confirmed cases for some countries. It is known that the continental ones tend to have higher numbers, both of cases and deaths. There are many factors that explain cases and deaths in a country such as the population density, percentage of the urban population, number of beds per hundred thousand inhabitants, human development index, Gini coefficient, poverty index, income per capita and life expectancy, the last one is the most important factor to yield higher death rates. It is also known, for example, that women are less likely than men to have fatal outcomes following coronavirus infection, although the reasons for this sex difference remain unclear.

The coronavirus pandemic had already affected more than 174 million people worldwide, leading to about 3.74 million deaths by 8 June 2021. Europe remains the continent most affected in the world by the coronavirus pandemic up to this date, with over 46.90 millions infected people and more than 1.08 million deaths [1]. It has 27% of the confirmed cases worldwide, and 29% of all deaths recorded globally, despite adding only 7.9% of the world population. The large proportion of deaths is due to high life expectancy, greater spread of the virus due to intense mobility, and several cities with very high population density.

Among the fourth-eight countries with death rates higher than 1000 deaths/million in the world (among 222 countries), there are seven in South America: Peru, Brazil, Colombia, Argentina, Chile, Bolívia and Ecuador. Brazil, on 8 June 2021, registered cases totaled 17 millions, its death toll is more than 476,000, and an overall lethality rate of 2.8%, while 15 million people have recovered from the virus so far according to data registered from Ministério da Saúde (Brazil) [2].

The pandemic came to Brazil through the arrival of foreigners and the return of Brazilians from Europe who disembarked at the ports and airports in Brazil, mainly those of São Paulo, Rio de Janeiro, Fortaleza, Recife and Salvador. It has gone from the rich to the most vulnerable poor people. Slum-dwellers, homeless people, drug users, elderly in nursing homes and riverine communities—and also from coastal cities to the interior—were all affected by the pandemic. It has reached devastatingly almost all 5568 Brazilian cities. For some time, the spread of the virus has been restricted in more affluent areas. However, the transmission has gradually been spread to cities further away from large urban centers, where their populations are more exposed to intense social vulnerability. Thus, it is not by chance that most Brazilian cities are suffering so much from the pandemic, as it is a continental country that suffers from health and social exclusion for a large number of poor people.

It is known that the North and Northeast regions in Brazil have the most vulnerable cities. These areas are the least developed ones, characterized by lots of populations with special needs—due to precarious health—an ageing population, and a high rate of poverty. São Paulo and Rio de Janeiro, such as cities in metropolitan regions and capitals of high population density states, are also marked by an increase of social vulnerability. The highest number of deaths from coronavirus occur in areas that have always suffered from poverty problems, such as lack of decent housing, water and sewage and soil pollution. The number of tests carried out for coronavirus in Brazil was quite small concerning the population size and it is still very low. The country indeed tested only 23% of the population, while the United States tested almost the whole population. As stated by the World Health Organization (WHO), the amount of cases reported is highly dependent on each country’s testing policy. In this matter, testing is crucial to understanding the spread of the pandemic and responding appropriately [3].

On 8th March 2021, Brazil began a 7-day moving average of more than 1500 deaths per day from coronavirus, which lead it to join the roll of the countries with the world’s worst death rates, alongside the USA and, later on, India. After that, Brazil started to show signs that the spread of the virus was easing in the country. The 7-day moving average of new confirmed cases has increased almost linearly since the beginning of November 2020 up to the end of March 2021 from 16 to 77 thousand per day, followed by a slight decrease to 62 thousand on 8 June. The 7-day moving average of coronavirus deaths in Brazil since 18 February 2021 has increased almost quadratic ally from 1030 to 3109 deaths per day on April 11 followed by a decrease to 1630 reached on 8 June. These statistics place the country as the third in the number of cases and second in terms of deaths in the world [1,2].

Besides lacking in doing tests, Brazil is one of the countries with the worst income distribution in the world [4]. In other words, the pandemic affected people who were already in expressive social vulnerability due to unemployment, poor housing conditions and difficulty in accessing health services. Under these circumstances, it is possible to identify which factors contribute to a fast spread of the virus: they are linked not only to their pathogenic characteristics but also to social and economic factors [5]. So, in this context, anyone can get the virus, but its impacts are not equal. However, low socioeconomic status is associated with low adherence to preventive measures as well—either through ignorance or having to take risks surviving [6].

Another fact to address is that the poorest people live in places with high population density, in small houses and with several people altogether, which facilitates the spread of the virus. In addition to that, they already suffer from a burden of other infectious pathologies, such as HIV/AIDS and tuberculosis, as well as chronic non-communicable diseases, like diabetes, hypertension and obesity, to mention some—these are risk factors for the worsening of the pandemic [7,8].

The main objective of this research is to present in detail the impacts of the coronavirus in Brazil and other countries in South America, and for Brazil to carry out more detailed analysis considering data from more than 5500 municipalities. In addition, this study aims to present, in links, applications with daily updates, as well as predictions using time series models for confirmed cases and deaths in Brazil from COVID-19.

According to [9], the COVID-19 mortality rates were different due to socioeconomic measures. The percentage differences in age-standardized mortality rates between the least and most disadvantaged census blocks were greater for COVID-19 mortality than for overall mortality, thus suggesting that the pandemic had a stronger impact on the most disadvantaged areas. Furthermore, people living in these census blocks experienced higher risks (absolute and relative) of dying. Searching topics through Google Trends as an indicator of public focus is very useful for decision-making and communication strategies for promoting public policies in a more transparent way [10].

The current values of the coronavirus incidence rates (per one hundred thousand inhabitants) in Brazilian states vary from 4173 cases in Maranhão (Northest Region) to 17,395 cases in Roraima (North Region), thus revealing a high regional heterogeneity [2]. The overall national incidence rate is about 8082 cases. From an overall perspective, the five regions of Brazil were affected at different levels: the highest numbers of cases were in the Southeast (37.5%) and Northeast (23.6%). In this scenario, it is crucial to consider some limitations when interpreting regional data because the underreporting of cases and deaths implies an underestimation of the data. It has not yet been possible to estimate the magnitude of underreporting and its impact on the estimates presented. However, there are several initiatives to model the underreporting aforementioned, based on very robust assumptions [11].

The coronavirus has become a major public health challenge in Brazil, and its behavior and impacts are still unknown. Thus, it is essential to improve the detection of cases that can be done with regularity, daily updating and transparency, so that the pandemic monitoring is efficient.

The rest of the paper is structured as follows. In Section 2, the incidence of coronavirus disease in Brazil is discussed, and the difficulties faced by the poorest, health workers and the devastating impacts on its economy. Section 3 presents all the data sources considered for the panoramic study of coronavirus in Brazil and other countries in South America. The computational library of the R programming language is also exported to obtain the death numbers and confirmed cases of COVID-19 for South American countries. Two web applications are also described to obtain information from coronavirus in Brazil, which are built based on an Application Programming Interface (API). In Section 4, general statistics on the coronavirus for Brazil and other South American countries are reported. Various statistics on the evolution of the pandemic are calculated and presented in the form of graphs, maps and tables. Several other statistics are also described at the state level, with an emphasis on the states of Rio de Janeiro and Pernambuco, which stand out with the highest lethality rates when compared to the other Brazilian states. In Section 5, the Generalized Additive Model for Location, Scale and Shape (GAMLSS) models are mentioned briefly to predict confirmed cases and deaths from the coronavirus and help control the pandemic in Brazilian states. Finally, in Section 6, some concluding remarks are addressed.

## 2. The Challenges of the Brazilian Unified Health System

Brazil has one of the fastest-growing coronavirus pandemic rates in the world, which caused the disease to be spread rapidly across the country. The social distance from the population and the isolation of the cases are essential to mitigate its progression. These factors, therefore, allowed time for the health services to be prepared for the increased need for hospitalizations, both in hospitals and Intensive Care Units (ICUs), consequently avoiding the collapse of the hospital care system [12,13]. The Unified Health System (SUS, acronym in Portuguese) of the Brazilian Ministry of Health guarantees access to health actions and services in this pandemic to the great majority of Brazilians. However, its management to combat the coronavirus was inefficient because it reached much higher levels than expected. About 94.4% of the individuals, who constitute the poorest 20% of the population, are dependent on the SUS.

The capacity of the SUS to perform its functions during the pandemic required not only the creation of new hospitals and ICUs but also a new operational organization within the health system, including the creation of new accesses, mainly by remote route [14]. So, in this context, all the modalities of call centers (consultation, counselling, monitoring and control of beds) have become vitally important in this process [15].

Indeed, remote patient care has proven to be an excellent tool in the fight against coronavirus and, at the same time, it reduced crowding in health services, making them safer and more efficient. In addition, patients with other pathologies can use remote care in the safety of their homes. In addition, despite all its problems, the SUS has been a vital piece in facing the pandemic. Now it becomes clear the need for more resources for the system to function well. In other words, the COVID-19 further exposed the problems of the unified health system, especially the uneven distribution of medium and high complexity infrastructure, as well as the limited ability to perform diagnostic tests.

### 2.1. Health Professionals at Risk

The pandemic is very dangerous for healthcare professionals due to frequent contact with the virus. Although there are no official data from Brazil, at the beginning of April, about 7000 health professionals had to leave work due to suspicious symptoms. On the front line of combating the pandemic, we have about 3.5 million health professionals working in hospitals and health units that are presented in 5570 Brazilian municipalities [16].

In regard to only nursing professionals, about 1.3 million technicians and 420 thousand nursing assistants work in Brazilian health units. However, these professionals are less paid and do not receive adequate assistance if they get infected with COVID-19. So, health professionals are in the daily struggle against the coronavirus. Among the factors that contribute to infection in them can be mentioned: an insufficient number of protective equipment, a large workload and a lack of rest [17]. Thus, they need adequate infrastructure with protective equipment, hours of rest, good food and psychological support. All this to reduce the risk of infection and ensure a little comfort in the workplace [18].

In addition to the insufficiency of hospitals and ICUs beds, several “field hospitals” were created with the hiring of professionals with precarious employment ties and without labor rights. In addition, many of these professionals were inexperienced and came from health courses that anticipated graduation to meet the demand [16]. In summary, it is necessary to recognize the importance of health professionals who work even under adverse conditions and at high risk.

### 2.2. Health Surveillance and Excess Mortality

In May 2020, the national household sample survey, which is carried out periodically in Brazil with data collection by telephone interview, included a module on COVID-19. This module has a continuous character; and approximately 48 thousand households are being interviewed per week, totaling about 193 thousand households per month, across the country. The sample from the beginning to the end of the survey is determined, and then the module will interview the same households during this period [2].

Data are being collected based on the Brazilian population, estimated at 210.1 million inhabitants. Of this total, it was estimated that, in May 2020, 24 million (11.4%) had flu symptoms and, of these, 20.2 million (84.3%) did not seek medical care, and 11.5 million (56.9%) took medication on their own. The medical prescription reported that 2.9 million patients and 526 thousand received visits from SUS teams with a greater concentration in the Northeast region of the country (43.7%). These data draw attention to the fact that the mortality rate may be lower, as many cases have not yet been confirmed by the absence of tests for COVID-19 [19].

Further, between March and May 2020, a study evaluated the excess of deaths in capitals and other municipalities of the federative units of Brazil, both in men and women. The results indicated an excess of 39,146 deaths for the period studied. This increase was greater among the capitals of the North, Northeast, and Southeast regions [20].

From that perspective, we can explain, at least partially, that the high number of deaths are related to undiagnosed coronavirus cases or by indirect causes. In other words, during the pandemic, many patients with different pathologies may neglect treatment for fear of seeking medical care or even for difficulties accessing health services that are prioritizing care for coronavirus cases.

### 2.3. The Coronavirus Economic Impact

Before the worsening of the pandemic, the prospects for the Brazilian economy were of an average growth for the Gross Domestic Product (GDP) of 2.5% for the biennium in 2019–2020. The impacts of the pandemic on the Brazilian economy were very heterogeneous. The most affected sector was services that represent 70% of the national GDP. It was followed by the industrial transformation sector, while the industrial sector agriculture continued to maintain positive growth.

The pandemic also impacted the Brazilian labor market. For now, it is not possible to estimate the duration of this impact or the resumption of economic activity. The recovery will likely occur oscillating due to the possibility of new measures of social isolation or the very cautious behavior of consumers and producers; and the immense heterogeneity of the sectors of economic activity. Uncertainty scenarios like this can lead to many companies not taking the risk of hiring labor, enduring a high unemployment rate.

The implementation of a wide range of measures from the Ministry of Economy has reduced the immediate negative impact of the pandemic on the country’s production, employment and income levels. Among these measures, the highlight was the expansion of the “Bolsa Família” Program; the Emergency Employment and Income Maintenance Benefit; Emergency Financial Aid for the population in need and informal workers; the Emergency Employment Support Program; and the expansion of resources and transfers to states and municipalities for health actions.

According to the Focus Bulletin, the projection for GDP in 2020 is down 4.7%, and, for inflation, as measured by the Broad Consumer Price Index (IPCA), the outlook is for an increase of 1.83% this year. The estimate is that the poverty rate in Brazil may increase by 6.5% due to the pandemic, affecting almost a quarter of the Brazilian population.

## 3. Data Sources

The data from the API maintained by the Brazil Project (Brazil.IO) were taken (https://brasil.io/home/ (accessed on 8 June 2021)) for calculating the statistics reported in the following sections. Brazil.IO is an accessible repository of public data that provides information on cases and deaths, obtained daily from the 27 state health departments in Brazil. Thus, data on coronavirus can be explored, stratified in cities, states and regions. The data in South America are obtained from government sources through the COVID-19 library (available on: https://cran.r-project.org/web/packages/COVID19 (accessed on 8 June 2021)), version 4.0.3 of the R programming language (available on: https://www.r-project.org/ (accessed on 6 June 2021)).

The R language (R Core Team 2021, [21]) through the version 4.0.3 was used for treatment and organization of the data, construction of indicators, graphs, maps and forecasts. In the Brazil Project (Brazil.IO) aforementioned, the API provides a national database with the historical series of confirmed cases and deaths by Brazilian municipality. This tool gives structured databases, thus allowing the analysis of statistics and building reliable and stable web applications. Based on these data, we developed a web application for Brazil, in constant evolution, that provides updated information automatically of COVID-19 at regional, state and municipal levels. The application can be accessed at: https://pedro-rafael.shinyapps.io/shinydashboard/ (accessed on 8 June 2021).

It is possible to interact with maps for Brazilian municipalities through this application. In the application, data are arranged according to the numbers of confirmed cases, confirmed deaths and lethality rates (in percentages) by regions in Brazil. The application also provides graphs of the historical series of COVID-19 (confirmed cases, deaths and lethality rates) for states and municipalities. Moving average plots for the series can also be constructed, and the user can choose the number of values to be used in smoothing. Like the Brazil.IO project, R’s COVID19 library, maintained by the COVID-19 Data Hub project (available on: https://covid19datahub.io/ (accessed on 8 June 2021)), provides a unified data set through data collection in trusted sources around the world, combining them with useful exogenous variables for a better understanding of the pandemic. More details on the Coronavirus Data Hub project can be found on the official website and also in [22]. The estimates reported in Section 5 were obtained from the Observatory of Respiratory Syndromes at the Statistics Department of the Federal University of Paraíba in Northeast Brazil, a project coordinated by one of the authors of this paper. On the project website http://shiny.de.ufpb.br/ (accessed on 8 June 2021) it is possible to obtain information on updated estimates at http://shiny.de.ufpb.br/st_pred/ (accessed on 8 June 2021). The estimates, available for municipalities and capitals, use GAMLSS models through the gamlss library in R package. Details of the GAMLSS and this library can be obtained from the book [23].

## 4. General Statistics

The database used to obtain COVID-19 information in Brazil consists of 2,061,303 records to 8 June 2021 from more than 5550 municipalities that notified at least one confirmed case in each city. The complete base is over and tends to increase significantly over time.

**Figure 1:**
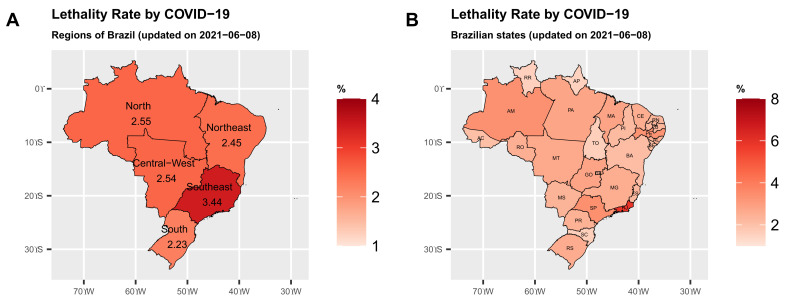
Lethality rate (%) by regions and states in Brazil (S are degrees South and W are degrees West).

Brazil consists of five geographic regions, North, Northeast, South, Southeast and Central-West. Among them, the Southeast region has the highest lethality rate (deaths/confirmed cases in percentage) of 3.44%, North (2.55%), Central-West (2.54%), Northeast (2.45%) and South (2.23%), respectively. Information regarding the number of confirmed cases, confirmed deaths and lethality rates by coronavirus in these regions until this date is presented in Table 1. The map of the demographic regions in Brazil is displayed in Figure 1.

Table 2 gives the lethality rates in ten Brazilian states ranked from the highest to the lowest. Note that of the ten states, seven have lethality rates higher than the national lethality rate of 2.8%, namely Rio de Janeiro (RJ) (5.84%), São Paulo (SP) (3.41%), Amazonas (AM) (3.35%), Pernambuco (PE) (3.28%) and Goiás (GO) (2.83%), respectively. For the COVID-19 mortality rates (deaths/residentend population per one million residents) in parentheses, the states of Mato Grosso (MT) (3190.95), Mato Grosso (AM) (3110) and Rio de Janeiro (RJ) (2986.55) have the highest mortality rates in Brazil.

**Figure 2:**
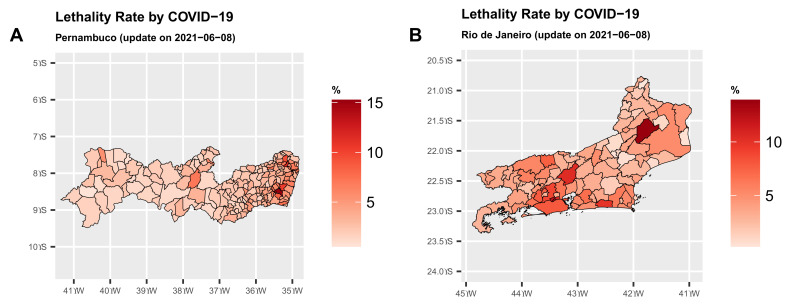
Map of the two states in Brazil with the highest lethality rate (%) (S are degrees South and W are degrees West).

Figure 1A clearly shows that the Southeast and North regions have the highest lethality rates by COVID-19 due to the states of Rio de Janeiro—RJ and Amazonas—AM, respectively. In the Northeast, the state of Pernambuco—PE, stands out with the highest lethality rate. Figure 2A,B show the maps with the lethality rates (in percentages) for these states. The lethality rate in the municipalities of the state of Rio de Janeiro—RJ is quite high in much of the state.

The municipalities in Pernambuco—PE with the highest rates, in general, are closer to the land-sea coastal border. In the state of Rio de Janeiro—RJ, 16% of the municipalities have a lethality rate above the state rate of 5.84%. On the other hand, 37% of the municipalities in Pernambuco—PE has a lethality rate above the state rate of 3.41%, which is a very worrying situation, although the number of confirmed cases has been declined in both states. For Pernambuco, 64.29% of the municipalities with populations above 100 thousand inhabitants have a lethality rate above the state rate. For the state of Rio de Janeiro—RJ, this percentage is close to 35.48%.

**Figure 3:**
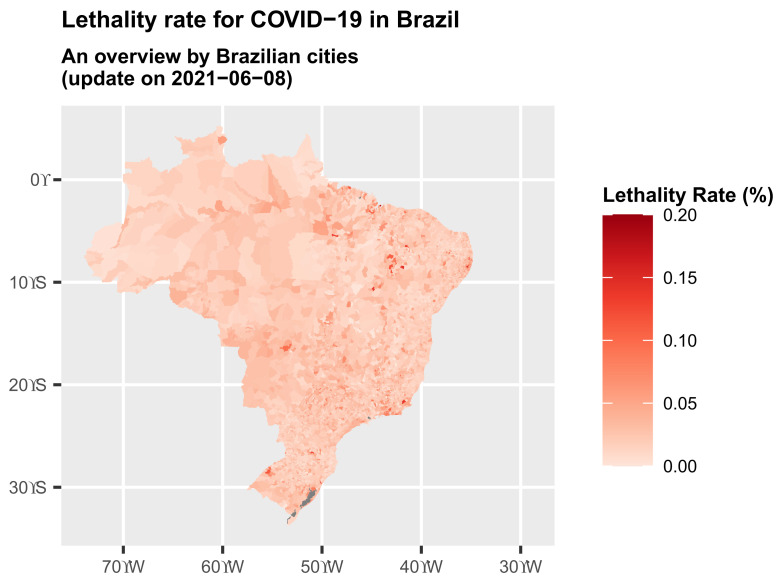
Map of an overview of the lethality (%) rate in Brazil by cities (S are degrees South and W are degrees West).

The coronavirus lethality rates in Brazilian municipalities can be seen on the map in Figure 3. From a general perspective, many of these municipalities have a lethality rate below 5%. More precisely, 95.02% of them have a lethality rate below 5%. It is noted that 71.84% of them have a lethality rate lower than the overall lethality rate of 2.8%, and 10.7% have this rate below 1%. The variances of the lethality rates for the North, Northeast, Southeast, South and Central-West regions are 0.5%, 0.15%, 2.66%, 0.29% and 0.09%, respectively, up to 8 June.

In regard to the absolute numbers of confirmed cases (17,042,198) and deaths (477,027) on 8 June by COVID-19, Brazil is the country in South America that stands out the most. In addition, Brazil, Argentina, Colombia and Chile have highlights when considering the absolute records of notified cases. This happens for obvious reasons, since Brazil, Argentina, Colombia and Chile have large populations corresponding to populations greater than 209, 444, 496 and 187 millions of inhabitants, respectively. In the case of Venezuela, it is clear that because it is a country with a closed regime, its statistics regarding confirmed cases and deaths may be far below the reality faced. The record of 2734 deaths and a lethality rate of less than 1%, more precisely 1.12%, does not seem consistent for a country with several serious problems in the areas of health, supply and the economy. Thus, a cautious look at Venezuela’s data is important.

Considering the percentage of lethality in the countries of South America, Brazil is in the position 4th of greater lethality. Countries such as Peru, Ecuador and Bolivia have a lethality percentage above Brazil, with lethalities of 9.4%, 4.81%, 3.88%, 2.8% and 2.66%, respectively; see Table 3.

**Figure 4:**
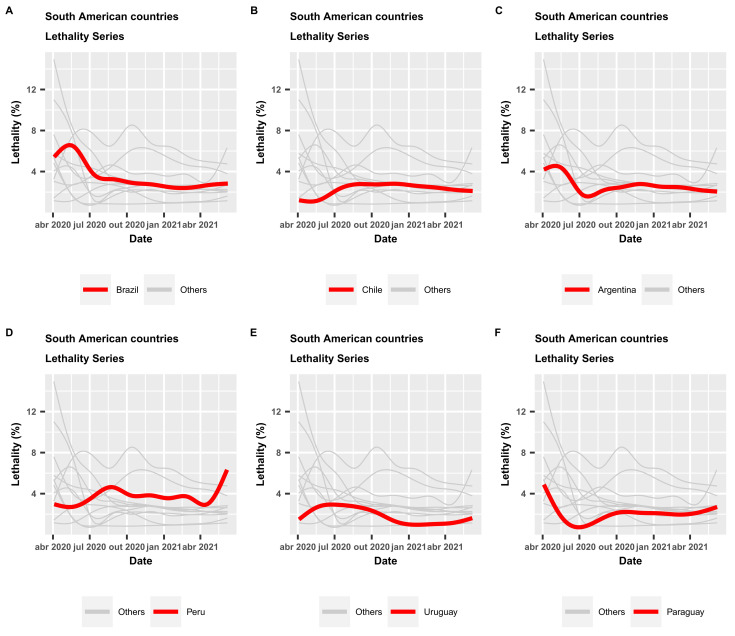
Time series of lethality rates (%) in South American countries.

**Figure 5:**
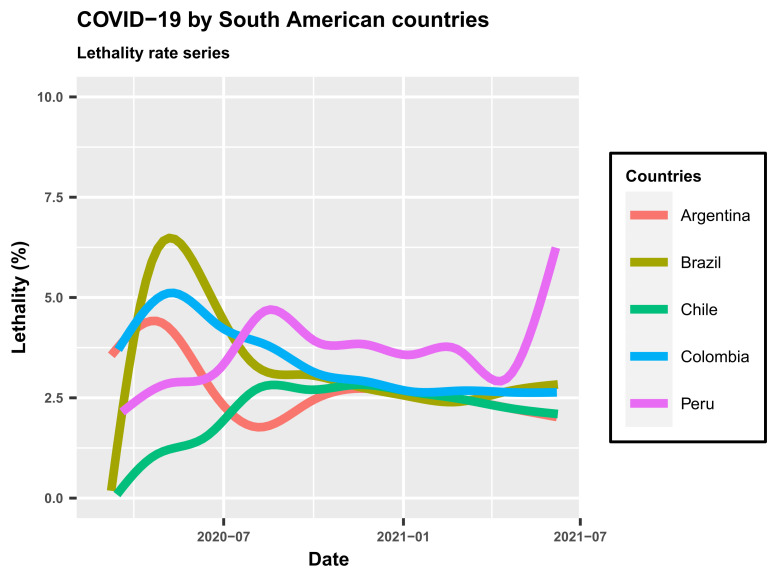
Series of lethality rates (%) for five countries in South America.

Figure 4A–F display the time series of the lethality rates for the countries of South America, with highlights (red lines) for those with great importance to the economy of this continent: Brazil, Chile, Argentina and Paraguay. The other lines (light gray) are the time series of the evolution of the lethality rate for the other countries of South America (except for French Guiana), thus allowing us to understand the situation of COVID-19 in South America. The time series of the mortality rates in these six countries can be compared from Figure 5. It is noted that from the beginning of April to the end of July 2020, Brazil occupied a prominent position with the highest lethality rate. Currently, other countries like Colombia and Peru are far worse than Brazil. The plots in Figure 5 show that Brazil is in a situation quite close to countries like Argentina and Chile, and only Peru is far above them.

## 5. Application of GAMLSS to Forecast Coronavirus in Brazil

The GAMLSS models are regression methods adopted in several studies [24]. We use GAMLSS models to provide forecasts about new cases and new deaths by coronavirus in the Brazilian states and their capitals. The GAMLSS modeling framework is currently implemented in a series of packages of the R statistical software (www.r-project.org (accessed on 8 June 2021)). The gamlss package allows to explain real data by considering more than fifty different distributions. For example, the exponential power distribution of Box–Cox [23] used by the World Health Organization to build the world standard growth curves [25], and the Poisson and negative binomial distributions. The last distribution is studied in several technical reports on coronavirus from the Imperial College COVID-19 Response Team in London [26].

It is considered that in the case of coronavirus, the growth of confirmed cases and deaths will necessarily decrease over time after some period. Therefore, it is justified that these confirmed cases and deaths registered over time can be fitted using a regression. Let $y={\left(y_{1},\cdots ,y_{n}\right)}^{⊤}$ be the response vector denoting the number of confirmed daily cases or the number of daily deaths. For this response variable, we consider the two-parameter logistic distribution, which is widely used to model population growth data. The choice of the logistic distribution to model the confirmed cases and deaths caused by coronavirus, via GAMLSS, is justified by the behavior of similar respiratory syndromes, which have a period of accelerated growth at the beginning of an epidemic and a slower period of decrease at the end of it that takes into account the logistic distribution, which is adequate for growth data.

### Application to Coronavirus Data

In this section, two GAMLSS regressions are chosen under the logistic distribution. The first regression considers the number of COVID-19 confirmed cases as a response variable, and the time as explanatory variable plus a dummy variable that corrects the effect of the under reporting identified on weekends. The historical series was considered from the reported 100th confirmed coronavirus case. The second regression follows the same structure to analyze the death data by considering the historical series from the 50th death recorded.

In order to provide forecasts related to new cases and new deaths caused by COVID-19 in Brazil, the UFPB Respiratory Syndromes observatory has developed web applications that can be accessed through the links http://obsrpb.com.br/ufpb/ (accessed on 8 June 2021) and http://shiny.de.ufpb.br/st_pred/ (accessed on 8 June 2021).

Based on data registered until 8 June 2021, it is possible to show evidence that new cases of COVID-19 are increasing in the following states: Alagoas, Bahia, Ceará, Maranhão, Mato Grosso do Sul, Mato Grosso, Minas Gerais, Paraíba, Paraná, Pernambuco, Piauí, Rio de Janeiro, Rio Grande do Norte, Roraima, São Paulo, Sergipe and Tocantins. By performing the predictions for the new confirmed cases in these states, there is evidence that the numbers of new cases are increasing, requiring a constant review of rules imposed on the population, as a way to prevent the transmission of the coronavirus.

**Figure 6:**
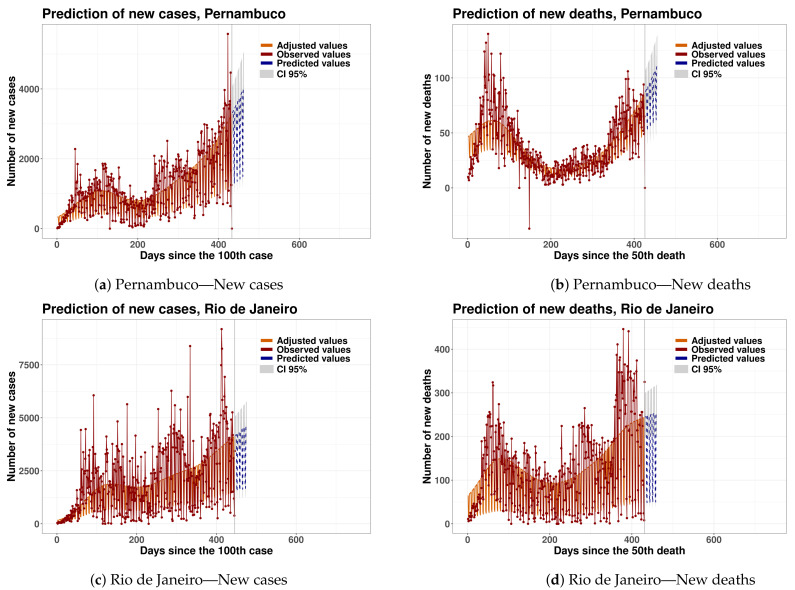
Time series and forecasts via GAMLSS models for new cases and new deaths in the next 30 days for the States of Pernambuco and Rio de Janeiro.

In relation to the state capitals, it is possible to show evidence that new cases of COVID-19 are increasing in the following capitals: Aracaju, Belo Horizonte, Boa Vista, Campo Grande, Palmas, Recife and Rio de Janeiro. Furthermore, when we analyze the data about new deaths, there is evidence that the number of new deaths are increasing in the following states: Alagoas, Bahia, Ceará, Goiás, Maranhão, Mato Grosso do Sul, Minas Gerais, Paraíba, Paraná, Pernambuco, Piauí, Rio de Janeiro, São Paulo and Sergipe. In relation to capital cities, there is the evidence that the number of new deaths by COVID-19 are increasing in the following capitals: Aracaju, Campo Grande, Curitiba, Palmas, Recife, Rio de Janeiro, Salvador, São Luís and São Paulo. Figure 6 shows the results described above for Pernambuco and Rio de Janeiro.

## 6. Conclusions

We reviewed the incidence of coronavirus disease in Brazil, which has had a devastating effect on the poorest population and on the country’s economy, highlighting the main difficulties of health workers. Brazil is a country of continental proportions and government authorities need accurate monitoring of confirmed cases and deaths from coronavirus. We built two web applications that constantly updates the evolution of cases and deaths in real time from COVID-19 data in the Brazilian states, their capitals and municipalities, totaling more than 5500 cities. All databases and sources for these applications used for monitoring the coronavirus pandemic in Brazil are described. We calculated useful statistics for coronavirus control and constructed interactive maps and graphs for confirmed cases, deaths and lethality rates. These applications are constantly evolving and have been used by health managers in some Brazilian states to guide public policies to combat the pandemic. We used the Generalized Additive Model for Location, Scale and Shape (GAMLSS) models to predict in short period confirmed cases and deaths from the coronavirus in order to assist government authorities in policies to combat the pandemic. 

## Figures and Tables

**Table 1:** COVID-19 statistics by regions of Brazil (update on 2021-06-08).

Regions	Cases	Deaths	Lethality Rate (%)
Central-West	1,757,326	44,693	2.54
North	1,635,038	41,741	2.55
Northeast	4,015,593	98,246	2.45
South	3,236,942	72,178	2.23
Southeast	6,392,230	219,934	3.44

**Table 2:** COVID-19 statistics for the ten Brazilian states with the highest lethality rate (update on 2021-06-08).

Date	State	Cases	Deaths	Population	Lethality Rate (%)	Mortality Rate Per Million
2021-06-08	Rio de Janeiro—RJ	888,367	51,865	17,366,189	5.84	2986.55
2021-06-08	São Paulo—SP	3,378,256	115,309	46,289,333	3.41	2491.05
2021-06-08	Amazonas—AM	390,386	13,086	4,207,714	3.35	3110.00
2021-06-08	Pernambuco—PE	502,697	16,468	9,616,621	3.28	1712.45
2021-06-08	Goiás—GO	623,443	17,616	7,113,540	2.83	2476.40
2021-06-08	Maranhão—MA	296,348	8365	7,114,598	2.82	1175.75
2021-06-08	Pará—PA	528,206	14,814	8,690,745	2.80	1704.57
2021-06-08	Mato Grosso—MT	419,855	11,252	3,526,220	2.68	3190.95
2021-06-08	Rio Grande do Sul—RS	1121,887	29,082	11,422,973	2.59	2545.92
2021-06-08	Ceará—CE	828,838	21,349	9,187,103	2.58	2323.80

**Table 3:** COVID-19 statistics by countries in South America.

Date	Country	Cases	Deaths	Population	Lethality Rate (%)	Mortality Rate Per Million
2021-06-06	Peru	1,983,570	186,511	31,989,256	9.40	5830.43
2021-06-07	Ecuador	432,739	20,814	17,084,357	4.81	1218.31
2021-06-07	Bolivia	387,162	15,024	11,353,142	3.88	1323.33
2021-06-08	Brazil	17,042,198	477,027	209,469,333	2.80	2277.31
2021-06-07	Paraguay	375,996	10,005	6,956,071	2.66	1438.31
2021-06-07	Colombia	3,593,016	92,496	49,648,685	2.57	1863.01
2021-06-07	Guyana	17,718	411	779,004	2.32	527.60
2021-06-07	Suriname	16,786	355	575,991	2.11	616.33
2021-06-08	Chile	1,440,417	30,104	18,729,160	2.09	1607.33
2021-06-07	Argentina	3,977,634	81,946	44,494,502	2.06	1841.71
2021-06-07	Uruguay	318,783	4692	3,449,299	1.47	1360.28
2021-06-07	Venezuela	243,621	2734	28,870,195	1.12	94.70

## Data Availability

Not applicable.

## Abbreviations

State	Acronym
Acre	AC
Alagoas	AL
Amapá	AP
Amazonas	AM
Bahia	BA
Ceará	CE
Espírito Santo	ES
Goiás	GO
Maranhão	MA
Mato Grosso	MT
Mato Grosso do Sul	MS
Minas Gerais	MG
Pará	PA
Paraíba	PB
Paraná	PR
Pernambuco	PE
Piauí	PI
Rio de Janeiro	RJ
Rio Grande do Norte	RN
Rondônia	RO
Roraima	RR
Santa Catarina	SC
São Paulo	SP
Sergipe	SE
Tocantins	TO
Distrito Federal	DF
Rio Grande do Sul	RS
